# Engineering Spatially Localized Bioactive Microenvironments in Assembly Implants for Spatially Extended Bone Ingrowth

**DOI:** 10.1002/advs.78161

**Published:** 2026-10-04

**Authors:** Heng Zhang, Jingjing Diao, Junhua Ke, Naru Zhao, Yudi Kuang, Yingjun Wang

**Affiliations:** ^1^ School of Materials Science and Engineering South China University of Technology Guangzhou People's Republic of China; ^2^ National Engineering Research Center For Tissue Restoration and Reconstruction South China University of Technology Guangzhou People's Republic of China; ^3^ Medical Devices Research & Testing Center of SCUT Guangzhou People's Republic of China; ^4^ Guangdong Institute of Advanced Biomaterials and Medical Devices Guangzhou People's Republic of China; ^5^ School of Biomedical Science and Engineering South China University of Technology Guangzhou International Campus Guangzhou People's Republic of China

**Keywords:** bioadaptability, diffusion‐confined calcium transport, osteogenic differentiation, segmental bone regeneration, spatially regulated microenvironment, Ti alloy/β‐TCP assembly implant

## Abstract

The repair of large segmental bone defects requires implants that combine structural stabilization with biological regulation. Here, we developed a bioadaptive modular implant integrating porous titanium (Ti) alloy for structural reinforcement with spatially localized β‐tricalcium phosphate (β‐TCP) modules for bioactive regulation. Using a gelatin methacryloyl (GelMA)‐based diffusion‐confined model, we identified spatial differences in β‐TCP‐associated calcium distribution and corresponding position‐dependent changes in hBMSC proliferation, adhesion, osteogenic differentiation, and Wnt/β‐catenin‐related signaling. The proximal region showing the strongest biological responses was used as a model‐derived spatial reference for organizing β‐TCP‐containing modules. In a rabbit femoral segmental defect model, the Ti alloy/β‐TCP assembly implant showed greater bone formation and ingrowth than porous Ti alloy controls. Histology further revealed that newly formed bone was preferentially associated with β‐TCP‐containing regions and extended into adjacent porous Ti structures. These findings demonstrate a proof‐of‐concept for translating experimentally characterized spatial biological responses into the spatial organization of structural and bioactive implant components.

## Graphical abstract

An assembly porous titanium/β‐TCP implant is developed to establish a spatially programmable osteogenic microenvironment for enhanced osseointegration. By decoupling mechanical support from bioactive ion release, localized ionic gradients generated from the β‐TCP module direct osteogenic activity and bone ingrowth toward the porous titanium framework, thereby promoting robust bone–implant integration and providing a new strategy for spatially functionalized bone implants.

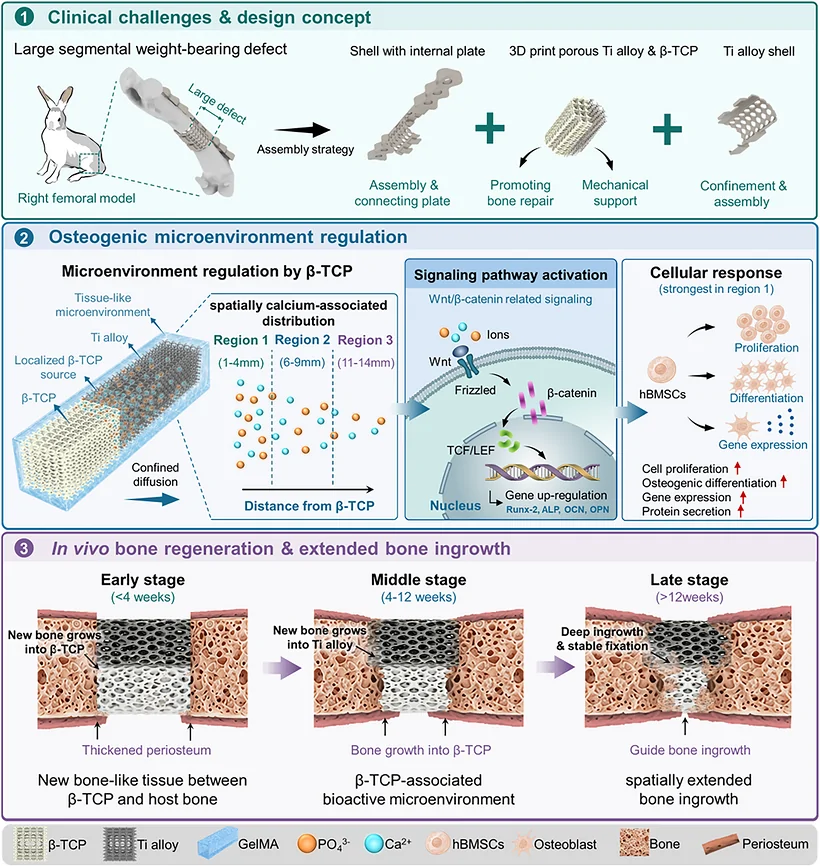

## 1 Introduction

Large‐size segmental bone defects caused by trauma, infection, and bone tumors, particularly in load‐bearing long bones, remain a significant clinical challenge due to the compromised osteogenic environment at the defect site and the high mechanical demands required for long‐term implantation [1, 2, 3, 4]. Autologous bone grafting is regarded as the gold standard for treating bone defects [5]; however, this approach requires harvesting bone from the patient, carrying potential risks such as donor site pain, infection, local numbness, and loss of structural integrity [6, 7, 8]. Furthermore, the limited availability of donor bone often fails to meet clinical needs [9]. Although traditional implant‐based repair methods have advanced limb‐salvage treatments, they are associated with complications such as stress shielding‐induced bone resorption and prosthesis loosening, which may even lead to fractures. With advancements in 3D printing technology, 3D‐printed bone repair implants can achieve precise reconstruction of bone tissue morphology [10, 11, 12, 13, 14, 15, 16]. In particular, 3D‐printed porous titanium (Ti) alloy implants have achieved remarkable clinical success in treating large‐size load‐bearing segmental bone defects, owing to their stable mechanical properties, matched elastic modulus, and personalized design capabilities. However, recent clinical studies indicate that these implants still face challenges in promoting osseointegration [17, 18]. They often exhibit insufficient bioactivity, a lack of regulatory capacity over the tissue microenvironment, and limited interaction and responsiveness with autologous tissues. Moreover, the inherently poor osteogenic environment within large segmental defects further delays bone regeneration [19, 20] and increases the risk of fibrous tissue infiltration, nonunion, and eventual implant failure [21, 22]. Therefore, successful regeneration of large segmental bone defects requires not only mechanical restoration but also the establishment of spatially organized biochemical signals capable of regulating cellular behaviors and tissue regeneration within the defect microenvironment.

To improve the biological performance of Ti implants, extensive efforts have been devoted to developing bioactive surface modification strategies. For example, Karthega et al. [23, 24] introduced micro/nanoscale porous structures on Ti surfaces through H_2_O_2_ treatment, facilitating hydroxyapatite deposition and improving surface bioactivity. Pätsi et al. [25, 26] fabricated TiO_2_ coatings using a sol–gel approach, achieving enhanced calcium‐phosphate deposition and improved bone–implant interfacial bonding. Similarly, Ong et al. [27] employed plasma spraying to deposit hydroxyapatite coatings onto Ti substrates, significantly improving implant bioactivity and interfacial adhesion. Beyond conventional coatings, recent studies have further introduced multifunctional Ti surfaces with antibacterial, immunomodulatory, and electromechanical regulation capabilities. For instance, Xue et al. [28] developed a Si/P/F‐doped TiO_2_ matrix with photothermal responsiveness, while Xu et al. [29] introduced a biomimetic triboelectric nanogenerator to activate mechano‐electro‐biochemical cascades for accelerating bone regeneration. These advances have substantially expanded the biological functions of Ti implants [30, 31, 32, 33, 34, 35]. However, some coating‐based systems may face challenges related to coating adhesion, interfacial stability, and long‐term functional persistence, depending on the coating composition, fabrication method, and loading environment [36]. These considerations motivate the exploration of complementary implant‐design strategies that integrate structural reinforcement with bioactive regulation throughout the three‐dimensional regenerative space [37, 38, 39, 40, 41, 42, 43, 44]. The regeneration of large segmental defects requires biological regulation extending throughout the defect space. During bone healing, cells are exposed to spatially and temporally dynamic biochemical signals, and their responses are influenced not only by the presence of regulatory factors but also by their local concentration, distribution, and persistence within the extracellular microenvironment. However, current implant designs rarely explicitly incorporate the spatial organization of biochemical cues within the regenerative space. Therefore, an important challenge is to develop implant systems that integrate structural reinforcement with spatially organized biological regulation. Bioactive ceramics capable of releasing calcium‐associated species provide a potential means of establishing such localized bioactive environments. Calcium is an essential component of bone mineralization and also participates in osteogenic regulation [45, 46, 47, 48] through interactions with cellular signaling pathways. Importantly, the spatial distribution of calcium‐associated species can be influenced by material degradation kinetics and transport constraints, potentially generating spatially heterogeneous biochemical environments [49, 50] around bioactive ceramic components. Nevertheless, how such spatial distributions are associated with heterogeneous osteogenic responses and how this information can be translated into implant design remain insufficiently understood.

In this study, we investigate a 3D‐printed porous Ti alloy/β‐tricalcium phosphate (β‐TCP) assembly implant for the reconstruction of large segmental femoral defects (as illustrated in Figure 1). The porous Ti alloy component provides structural reinforcement within the plate‐stabilized defect, whereas the β‐TCP component serves as a spatially localized osteoconductive and bioactive module. A simplified gelatin methacryloyl (GelMA)‐based diffusion‐confined model was first used to characterize spatial differences in β‐TCP‐associated calcium distribution and position‐dependent cellular responses. The proximal region exhibiting the strongest and most consistent biological responses was then used as an empirical spatial design reference for organizing β‐TCP‐containing modules within the implant. We subsequently evaluated the regenerative performance and spatial pattern of bone formation in a rabbit femoral segmental defect model. Rather than establishing a universal ionic threshold or an independently optimized spatial configuration, the present study provides a proof‐of‐concept for translating experimentally observed spatial biological responses into modular implant design.

**FIGURE 1:**
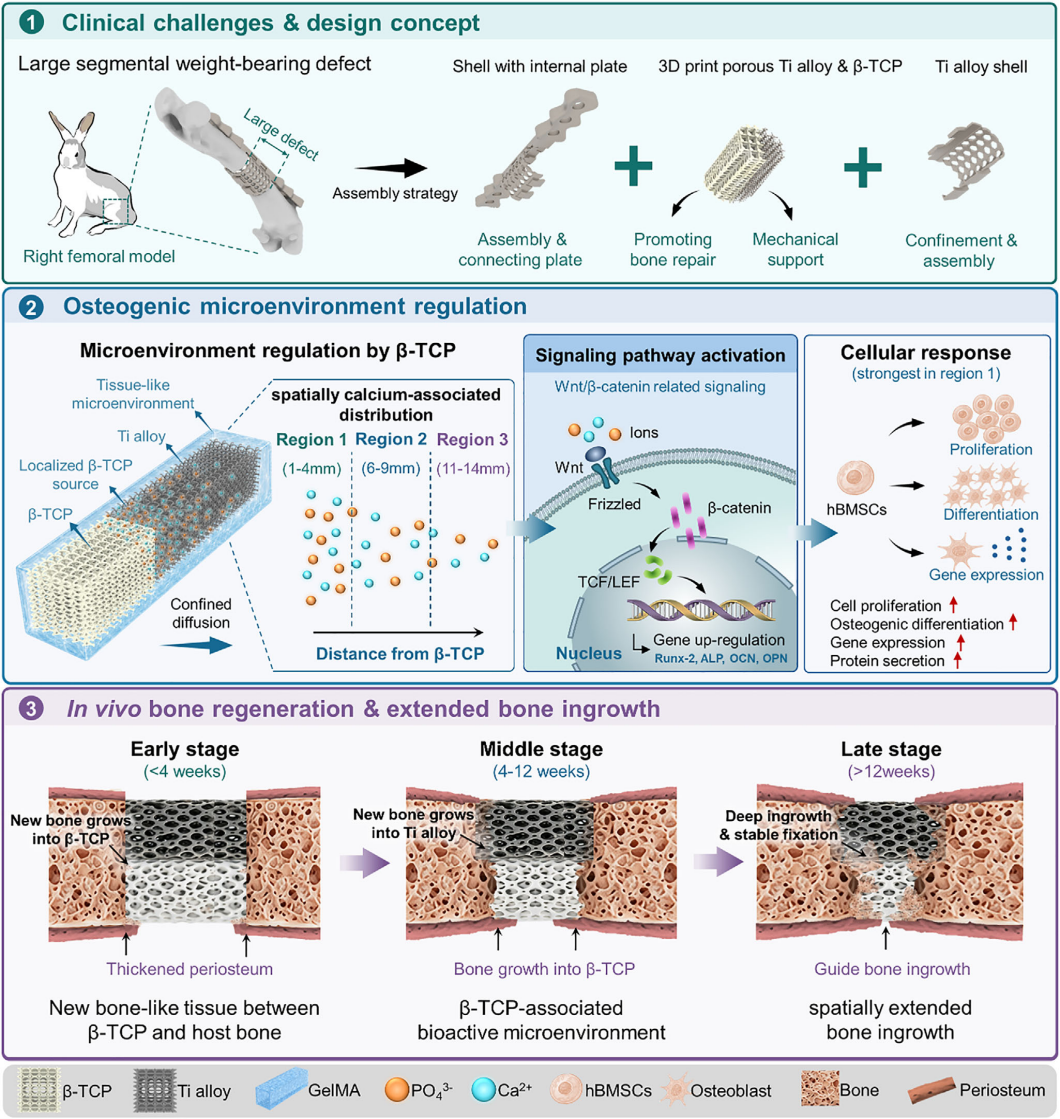
Schematic illustration of the study concept and experimental workflow. **Design concept**: A modular assembly strategy combining 3D‐printed porous Ti alloy and β‐TCP is developed for reconstruction of a large segmental bone defect, with the Ti alloy component providing structural reinforcement and the β‐TCP component providing spatially localized bioactivity. ***In vitro* spatial model**: A GelMA‐based diffusion‐confined system is used to characterize β‐TCP‐associated calcium distribution and position‐dependent hBMSCs responses, including Wnt/β‐catenin‐related signaling involvement. ***In vivo* outcome**: The model‐derived spatial information is incorporated into the modular implant architecture, and bone formation and bone ingrowth are evaluated in a rabbit femoral segmental defect model.

## 2 Results and Discussion

### 2.1 Diffusion‐confined Spatial Distribution of β‐TCP‐Associated Calcium

To establish a controllable three‐dimensional environment for examining spatial transport from a localized β‐TCP source, a 5% (w/v) GelMA hydrogel was used as a simplified extracellular matrix (ECM)‐inspired diffusion‐confined matrix. The hydrated polymer network was intended to suppress bulk convection and slow rapid solute homogenization while remaining permissive for human bone marrow mesenchymal stem cells (hBMSCs) encapsulation and diffusion‐mediated transport. The 5% formulation provided sufficient structural integrity for maintaining the three‐dimensional experimental system and exhibited a tensile strength of 87.2 kPa, a shear modulus of 394.4 Pa, and an elastic modulus of 378.6 Pa under the present experimental conditions (Figure S1). These measurements characterize the physical environment used in the present model but are not intended to establish quantitative equivalence with native bone extracellular matrix.

Within the diffusion‐confined GelMA matrix, β‐TCP‐derived calcium exhibited a spatially heterogeneous distribution that evolved over time. Alizarin Red S (ARS) staining showed greater calcium‐associated staining near the β‐TCP source at the early time point, followed by progressive extension into the surrounding hydrogel (Figure 2a). Because ARS does not distinguish soluble Ca^2^^+^ from bound or precipitated calcium‐containing material, these observations were interpreted qualitatively as evidence of spatially heterogeneous calcium‐associated distribution. Inductively coupled plasma optical emission spectrometry (ICP‐OES) analysis further revealed distance‐ and time‐dependent differences in total elemental calcium among the predefined sampling regions. At day 7, the total calcium concentration reached 102.64 ppm in region 1, compared with 58.35 ppm in region 2 and 22.88 ppm in region 3 (Figure 2c). Because ICP‐OES quantifies total elemental calcium rather than local free Ca^2^^+^ activity, these measurements were used to characterize β‐TCP‐associated spatial calcium heterogeneity rather than the complete ionic environment experienced by the cells.

**FIGURE 2:**
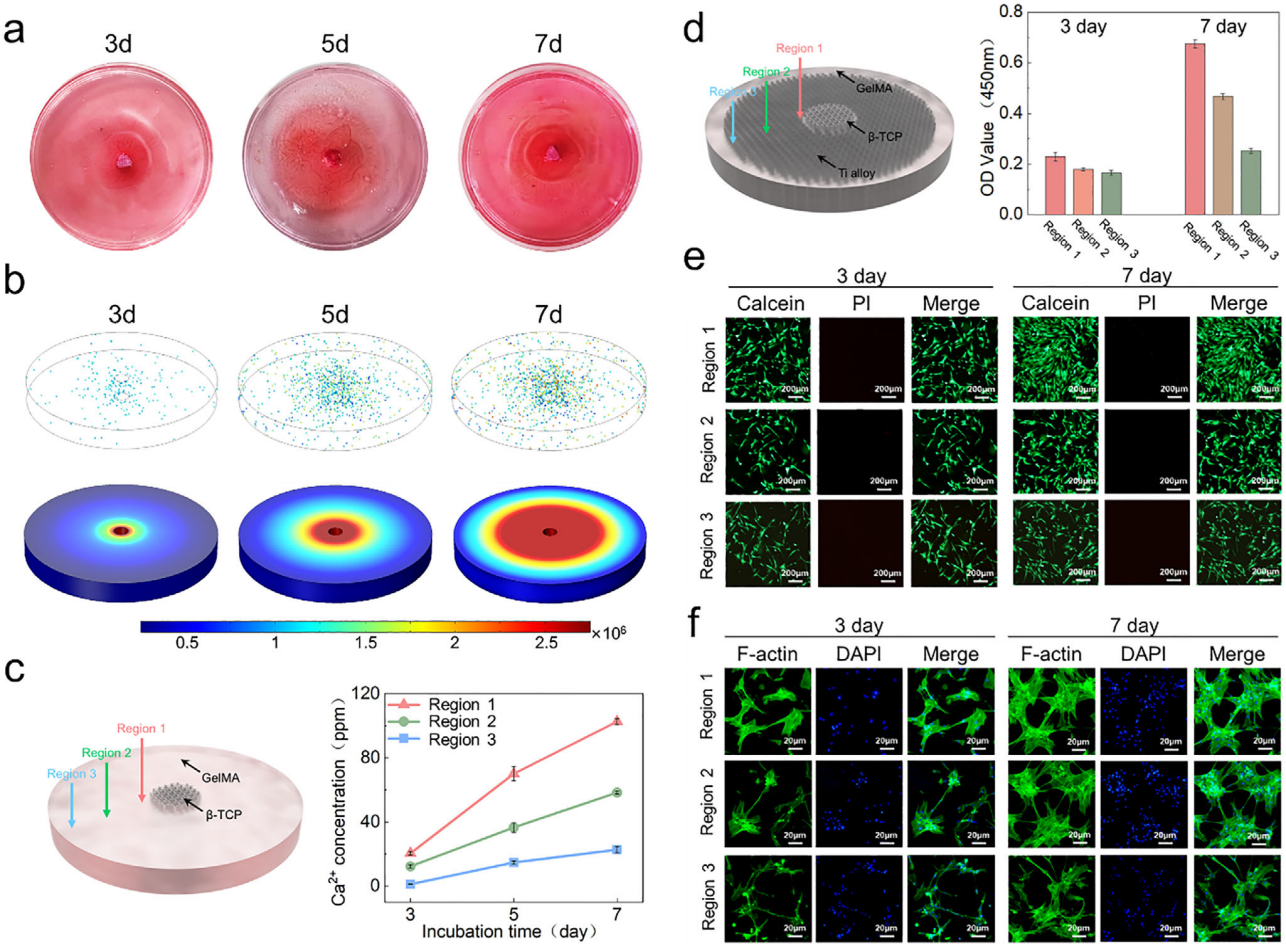
Spatial calcium‐associated distribution and position‐dependent hBMSCs responses in a 3D ECM‐inspired culture system. (a) Qualitative visualization of calcium‐associated spatial distribution in the GelMA matrix at 3, 5, and 7 days. (b) FEA illustrating the spatial attenuation tendency of diffusion from the localized β‐TCP source. (c) Schematic of the predefined sampling regions and total elemental calcium concentrations determined by ICP‐OES. (d) CCK‐8 analysis of hBMSCs in different spatial regions at 3 and 7 days. (e) Calcein‐AM/PI staining of hBMSCs in different regions at 3 and 7 days. (f) F‐actin/DAPI staining showing position‐dependent cell spreading and cytoskeletal organization. Statistical comparisons were performed independently at each time point using one‐way ANOVA followed by Tukey's multiple‐comparison test. Data are presented as mean ± SD, *n* = 6. ^*^ *p* < 0.05, ^**^ *p* < 0.01, and ^***^ *p* < 0.001.

Finite element analysis (FEA) was further performed to visualize the expected spatial attenuation of diffusion from the localized β‐TCP source (Figure 2b). The simulated distribution exhibited a spatial attenuation pattern qualitatively consistent with the experimentally observed calcium‐associated distribution and was used as a simplified representation of diffusion behavior.

Collectively, these findings demonstrate that the GelMA‐based system established a diffusion‐constrained, spatially heterogeneous calcium‐associated distribution around the localized β‐TCP source. These observations provide a physicochemical context for comparison with the position‐dependent cellular responses examined in subsequent experiments.

### 2.2 Position‐Dependent Cellular Responses Within a β‐TCP‐Associated 3D Microenvironment

To determine whether hBMSCs behavior varied according to position relative to the localized β‐TCP source, cell proliferation and adhesion were assessed across predefined regions of the GelMA matrix. A position‐dependent trend in cellular activity was observed across all assays. Cell proliferation, quantified by Cell Counting Kit‐8 (CCK‐8) and Calcein‐AM/PI staining, was consistently highest in region 1 (1–4 mm from the β‐TCP source), followed by region 2 (6–9 mm) and region 3 (11–14 mm) (Figure 2d,e). A similar spatial hierarchy was observed at both day 3 and day 7, with region 1 exhibiting the highest proliferative activity among the predefined regions.

A similar spatial dependence was observed in cell adhesion and cytoskeletal organization. F‐actin/DAPI staining showed that cells in the proximal region exhibited greater spreading, more developed actin stress fibers, and more pronounced pseudopod extension, whereas cells in the distal regions remained relatively rounded and displayed less organized cytoskeletal structures (Figure 2f). Quantitative analysis confirmed a progressive decrease in cell spreading area with increasing distance from the β‐TCP source (Figure S2). The consistency between proliferation and adhesion responses indicates that the spatially heterogeneous β‐TCP‐associated microenvironment was associated with both early adhesion behavior and subsequent proliferative activity.

Collectively, these results demonstrate position‐dependent cellular heterogeneity within the β‐TCP‐associated 3D microenvironment, with the strongest proliferative and spreading responses consistently observed in the proximal region. This spatial pattern broadly corresponded to the calcium‐associated distribution characterized in Section 2.1 and was interpreted as a spatial association. These findings provided the basis for further examining whether osteogenic responses exhibited a similar position‐dependent pattern.

### 2.3 Position‐Dependent Osteogenic Responses and Involvement of Wnt/β‐Catenin‐Related Signaling

To determine whether osteogenic responses varied according to spatial position relative to the localized β‐TCP source, osteogenesis‐related gene expression of hBMSCs was analyzed across the predefined hydrogel regions. A pronounced position‐dependent osteogenic response was observed, with region 1 consistently exhibiting the highest expression of Runx2, ALP, OCN, and OPN at both 7 and 14 days, followed by progressively lower expression in regions 2 and 3 (Figure 3a). These findings demonstrate spatially heterogeneous osteogenic responses associated with proximity to the localized β‐TCP source.

**FIGURE 3:**
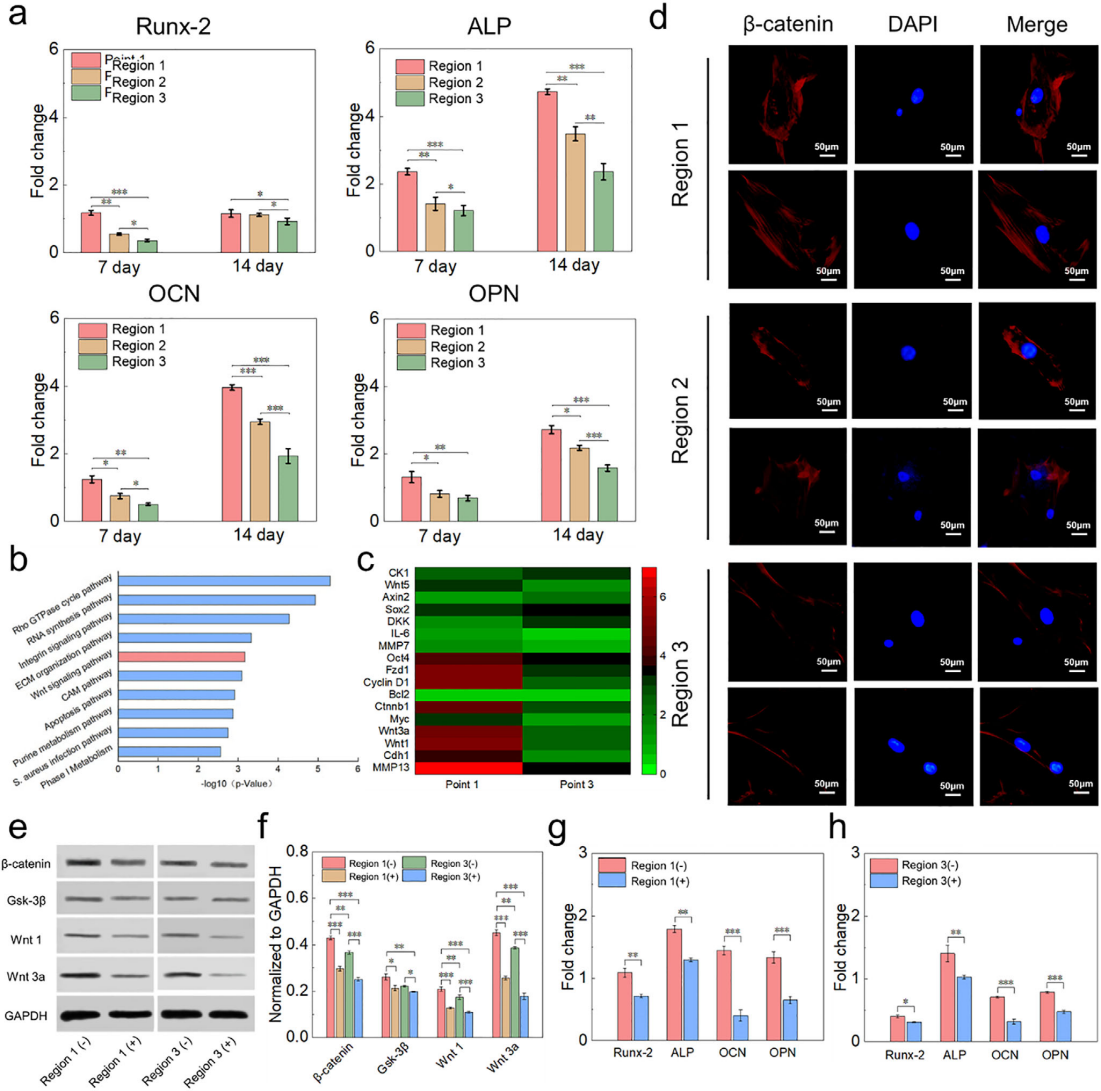
Position‐dependent osteogenic responses of hBMSCs and involvement of Wnt/β‐catenin‐related signaling within the β‐TCP‐associated 3D microenvironment. (a) Expression of osteogenesis‐related genes in hBMSCs cultured in different regions of the 3D matrix for 7 and 14 days. (b) Differentially regulated signaling pathways identified by transcriptomic analysis of hBMSCs collected from different spatial regions. (c) Heatmap of differentially expressed genes associated with Wnt/β‐catenin‐related signaling across the different spatial regions. (d) Immunofluorescence staining of β‐catenin (red) and DAPI (blue) in different regions of the 3D culture matrix. (e) Western blot analysis of β‐catenin‐related proteins following pathway inhibition with XAV939. (f) Quantification of Western blot band intensities. (g) Expression levels of osteogenesis‐related genes in region 1 with or without XAV939 treatment. (h) Expression levels of osteogenesis‐related genes in region 3 with or without XAV939 treatment. Statistical comparisons were performed independently at each time point using one‐way ANOVA followed by Tukey's multiple‐comparison test, whereas comparisons between XAV939‐treated and untreated groups were performed using an unpaired two‐tailed Student's *t*‐test. Data are presented as mean ± SD, *n* = 6. ^*^ *p* < 0.05, ^**^ *p* < 0.01, and ^***^ *p* < 0.001.

To elucidate the underlying molecular mechanism, transcriptomic profiling was performed on hBMSCs collected from the different spatial regions. Pathway enrichment analysis revealed that Wnt/β‐catenin signaling was among the most significantly regulated pathways among cells sampled from different spatial regions (Figure 3b). Heatmap analysis further confirmed coordinated upregulation of Wnt‐associated genes (Figure 3c), including key transcriptional mediators such as TCF and LEF in region 1 (Figure S3). Consistently, immunofluorescence analysis demonstrated enhanced β‐catenin accumulation and nuclear localization in the proximal β‐TCP‐associated region, supporting greater involvement of Wnt/β‐catenin‐related signaling in this region (Figure 3d).

To further examine the involvement of Wnt/β‐catenin signaling, pathway inhibition was performed using XAV939. Suppression of β‐catenin signaling significantly reduced osteogenic gene expression in the examined regions. Specifically, XAV939 treatment reduced the expression of Runx2, ALP, OCN, and OPN in region 1, with the maximum reduction reaching 74.2% (Figure 3e–h).

Collectively, these findings demonstrate that hBMSCs exhibited position‐dependent osteogenic responses within the β‐TCP‐associated 3D microenvironment, with the strongest osteogenic activity observed in the proximal region. The corresponding spatial differences in Wnt/β‐catenin‐related signaling, together with the inhibitory effects of XAV939, support the involvement of this pathway in the observed osteogenic response.

### 2.4 Model‐Derived Spatial Reference for Modular Implant Design

The biological responses within the three‐dimensional GelMA system exhibited clear spatial differences relative to the localized β‐TCP source. Across the predefined sampling regions, region 1 (1–4 mm) consistently showed higher proliferative activity, greater cell spreading, and stronger osteogenic responses than the more distal regions 2 (6–9 mm) and 3 (11–14 mm). The expression of osteogenic markers, including Runx2, ALP, OCN, and OPN, followed a similar spatial pattern. Under the simplified transport conditions, higher total calcium levels were also detected in region 1. Collectively, these findings identified the proximal region as the spatial domain exhibiting the strongest and most consistent biological responses under the present experimental conditions.

To translate this spatial information into implant design, the outer boundary of region 1 (∼4 mm) was operationally used as a model‐derived spatial design reference (Figure S4). This value corresponds to the outer boundary of the predefined proximal region in which multiple biological readouts consistently exhibited the strongest responses, rather than to a prospectively defined biological cutoff or a directly measured free‐ion diffusion distance. Accordingly, the approximately 4‐mm value was used as a system‐specific empirical design‐guiding scale for organizing β‐TCP‐containing modules within the investigated implant configuration.

Based on this model‐derived spatial reference, the investigated Ti alloy/β‐TCP assembly implant was configured such that the adjacent porous Ti alloy regions were positioned within approximately 4 mm of the β‐TCP‐containing modules (Figure 4a and Figure S5). This configuration was intended to examine whether spatial biological information obtained from the simplified in vitro model could be translated into the organization of structurally and biologically distinct implant modules. Importantly, this approximately 4‐mm scale was used to guide, rather than rigidly determine, the implant architecture.

**FIGURE 4:**
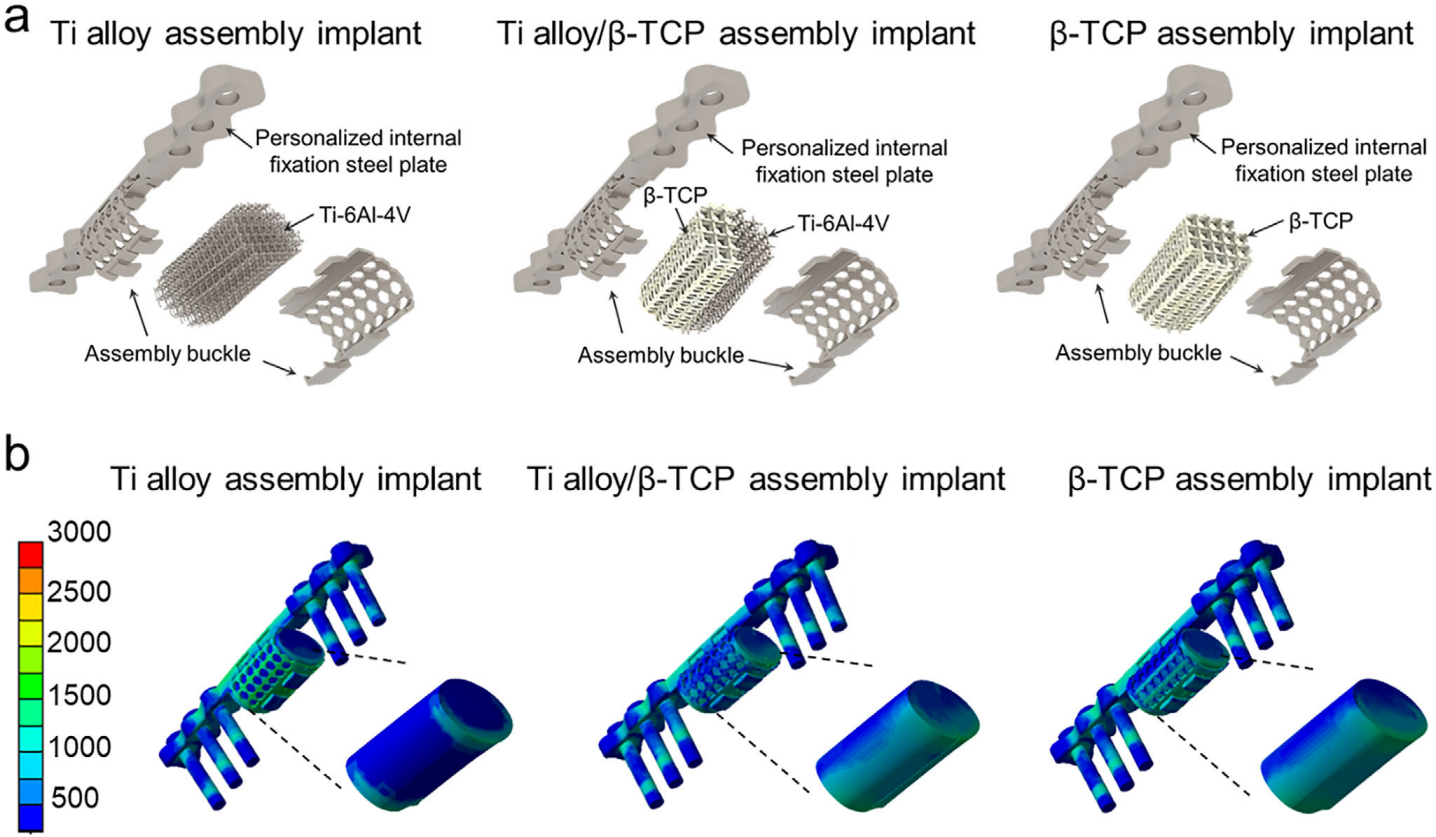
Design and finite element analysis of the 3D‐printed modular implants. (a) Schematic illustration of the modular implant configurations. (b) Finite element analysis showing stress distributions of the different implant configurations under the simulated loading condition.

Finite element analysis further showed that the assembled configuration maintained a comparatively uniform stress distribution under the prescribed loading conditions, whereas the standalone β‐TCP configuration exhibited greater localized stress accumulation (Figure 4b). These findings indicate that the incorporation of β‐TCP modules within the porous Ti alloy architecture preserved a mechanically reinforced configuration under the simulated conditions.

More broadly, the significance of this approach lies not in the absolute 4‐mm value itself, but in the design principle of experimentally characterizing spatial biological responses around a localized bioactive component and subsequently translating this information into the spatial organization of a modular implant.

### 2.5 Spatially Extended Bone Ingrowth in Ti Alloy/β‐TCP Assembly Implants

A large femoral segmental defect model in New Zealand white rabbits was established to evaluate the regenerative performance of the 3D‐printed Ti alloy/β‐TCP assembly implant (*n* = 5) compared with porous Ti alloy and porous β‐TCP controls. Customized surgical guidance facilitated reproducible defect creation, implant positioning, and implant–host bone alignment (Figures S6–S8). Among the specimens that reached the scheduled 12‐week endpoint, radiographic and gross observations showed maintained implant positioning and fixation without evident displacement of the analyzed constructs (Figure S9). Micro‐CT analysis showed progressive mineralized tissue formation within the defect region from 4 to 12 weeks. The Ti alloy/β‐TCP assembly group exhibited a higher Micro‐CT‐derived BV/TV than the porous Ti alloy group at the investigated time points (Figure 5a,b). Because residual β‐TCP and newly mineralized bone exhibit partially overlapping attenuation characteristics, these BV/TV values are interpreted as standardized estimates of mineralized bone volume and are considered together with the corresponding undecalcified histological findings.

**FIGURE 5:**
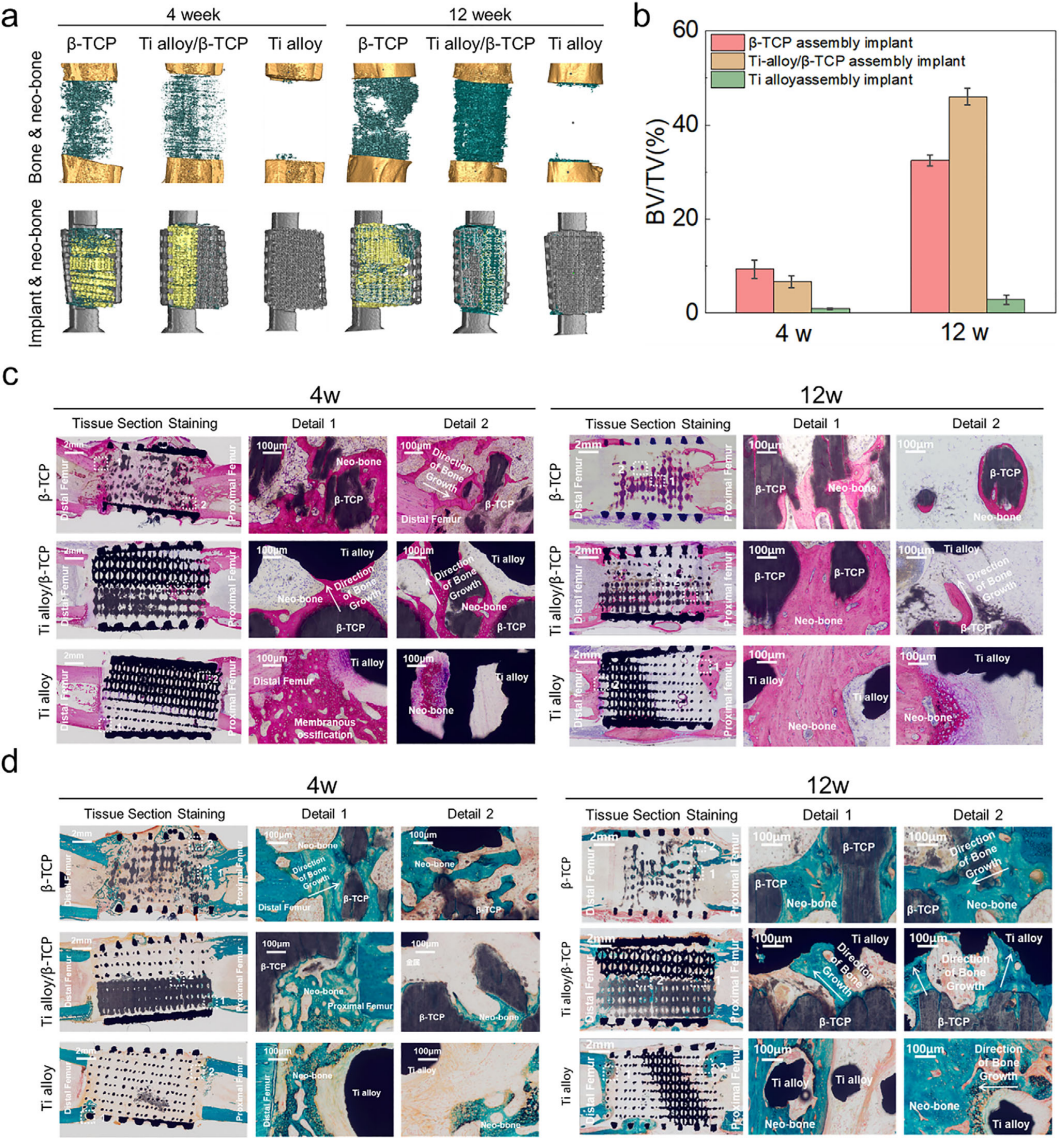
Histological and micro‐CT analysis of implants in large‐size segmental femoral defects at 4‐ and 12‐weeks post‐implantation. (a) Micro‐CT reconstruction at 4‐ and 12‐weeks post‐implantation. (b) BV/TV ratios at 4 weeks and 12 weeks post‐implantation. (c) MB‐BF staining at 4 and 12 weeks post‐implantation. (d) Masson staining at 4 and 12 weeks post‐implantation. Statistical comparisons were performed independently at each time point using one‐way ANOVA followed by Tukey's multiple‐comparison test. Data are presented as mean ± SD, *n* = 5. ^*^ *p* < 0.05, ^**^ *p* < 0.01, and ^***^ *p* < 0.001.

Histological analysis using Methylene blue/basic fuchsin (MB/BF) and Masson's trichrome staining further demonstrated differences in the spatial pattern of bone formation among the implant configurations (Figure 5c,d). At 4 weeks, early osteoid deposition in the assembly implant was predominantly observed around β‐TCP‐containing regions. With increasing implantation time, newly formed bone progressively occupied the β‐TCP‐containing regions and extended into adjacent porous Ti alloy regions. By 12 weeks, substantial bone ingrowth was observed across the β‐TCP/Ti interfaces and within adjacent porous Ti regions. Quantitative histomorphometry showed a greater new‐bone area in the assembly implant (Figure S10), with additional histological views provided in Figures S11 and S12. Representative histological observations of both the 30/70 and 70/30 Ti alloy/β‐TCP configurations showed a similar spatial pattern, with newly formed bone associated with β‐TCP‐containing regions and extending into adjacent porous Ti structures (Figure S13a–d). Because the adjacent Ti regions in the 30/70 configuration were only approximately 2–3 mm in length, the measurable extension distance was geometrically constrained and was therefore not suitable for direct quantitative comparison with the 70/30 configuration. Quantitative analysis was consequently performed for the 70/30 configuration, in which sufficient adjacent Ti length was available for spatial measurement. The measured bone‐extension distance was approximately 3–4 mm at 12 weeks (Figure S13e). This value showed an empirical correspondence with the approximately 4‐mm model‐derived spatial reference obtained from the in vitro GelMA system, but should not be interpreted as validation of an invariant biological regulatory distance.

An exploratory 50/50 Ti alloy/β‐TCP configuration with an alternative spatial arrangement showed a qualitatively similar regenerative pattern, with newly formed bone associated with β‐TCP‐containing regions and extending toward adjacent porous Ti structures (Figure S14). Because this configuration was not prospectively matched to the primary configurations for composition, geometry, interface area, or mechanical properties, it is presented only as exploratory evidence of a spatially associated regenerative pattern rather than as a causal control.

Immunohistochemical analysis further revealed spatial differences in osteogenic activity within the assembly implant (Figure 6a,b). ALP and β‐catenin expression was higher in regions adjacent to β‐TCP‐containing modules and decreased toward more distal porous Ti regions. Together with the histological findings, these results demonstrate that bone formation and osteogenic activity were spatially associated with the location of β‐TCP‐containing regions within the modular implant.

**FIGURE 6:**
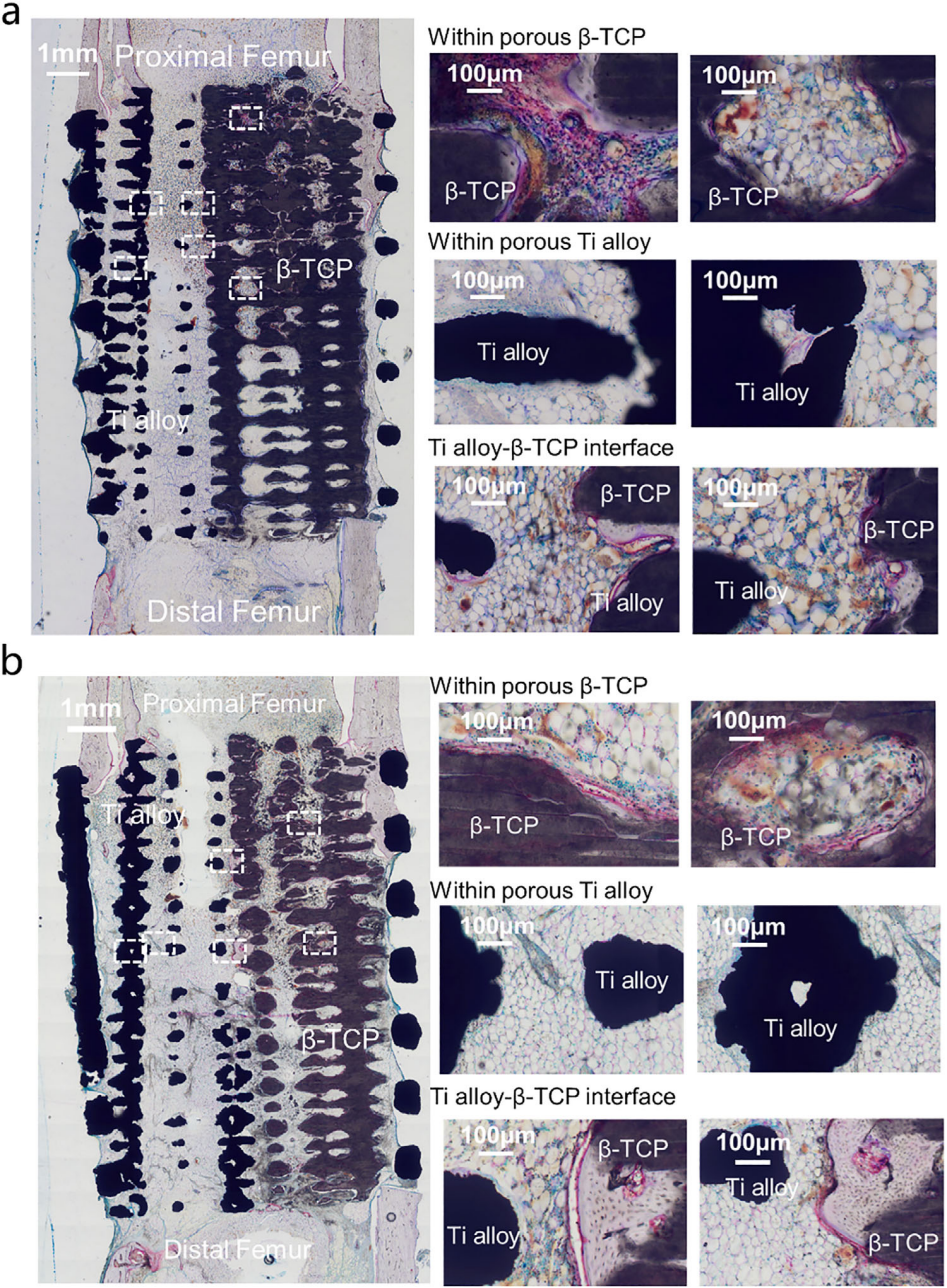
Immunohistochemical analysis of implants in large‐size segmental femoral defects at 8 weeks post‐implantation. (a) ALP immunohistochemical staining of the assembly implant. (b) β‐catenin immunohistochemical staining of the assembly implant.

Notably, the porous β‐TCP group did not exhibit the greatest overall bone regeneration despite its higher ceramic content. Although β‐TCP provided an osteoconductive environment and was associated with active local bone formation, progressive degradation and fragmentation of the β‐TCP‐only construct were accompanied by loss of structural continuity and heterogeneous defect bridging. In contrast, the Ti alloy/β‐TCP assembly combined β‐TCP‐associated osteoconductive and bioactive functions with the structural reinforcement provided by the porous Ti alloy component under plate‐stabilized conditions. This combination maintained an interconnected porous architecture while preserving localized β‐TCP‐associated bioactivity and was associated with greater overall bone formation and more extensive bone ingrowth across the defect. These observations suggest that increasing the amount of β‐TCP alone does not necessarily translate into greater overall regeneration; rather, the regenerative outcome likely depends on the integration of bioactivity with structural and spatial characteristics of the implant.

Overall, the Ti alloy/β‐TCP assembly implant exhibited greater bone formation and spatially extended bone ingrowth than the porous Ti alloy and porous β‐TCP controls under the investigated plate‐stabilized conditions. Bone formation was preferentially associated with β‐TCP‐containing regions and extended into adjacent porous Ti regions, accompanied by higher ALP and β‐catenin expression near the β‐TCP‐containing modules. These findings support the feasibility of using model‐derived spatial biological information to organize localized bioactive ceramic modules within a structurally reinforced implant architecture for large segmental bone repair.

### 2.6 Future Perspectives and Limitations

From a translational perspective, the modular architecture provides a flexible framework for integrating structural reinforcement and localized bioactivity within patient‐specific implants. Because the Ti alloy and β‐TCP components can be independently designed and manufactured, their geometry, proportion, and spatial organization may be adapted to defect anatomy and regenerative requirements. More importantly, the conceptual advance of the present study lies in using experimentally characterized spatial biological responses as a design input for organizing structurally and biologically distinct implant modules, rather than relying solely on the amount of bioactive material incorporated. This model‐informed strategy provides a potential framework for translating spatial biological information into implant architecture.

Several limitations should nevertheless be considered. First, the approximately 4‐mm spatial reference was derived from the outer boundary of the proximal region showing the strongest and most consistent biological responses in the present GelMA model and should therefore be regarded as a system‐specific empirical design reference rather than a universal biological threshold. In addition, the physicochemical characterization primarily reflected β‐TCP‐associated total calcium distribution under simplified transport conditions and did not comprehensively resolve local free Ca^2^^+^ activity, phosphate species, pH, or β‐TCP degradation kinetics under the actual cell‐culture environment. The apparent correspondence between this model‐derived reference and the approximately 3–4 mm bone extension observed in the 70/30 Ti alloy/β‐TCP configuration should therefore be interpreted as an empirical spatial association that requires further validation under more physiologically representative conditions.

Second, the present in vivo comparisons were not designed to isolate β‐TCP spatial distribution as an independent variable. Differences among the porous Ti alloy, porous β‐TCP, and Ti alloy/β‐TCP implants involved multiple coupled factors, including material composition, structural characteristics, degradation behavior, and spatial organization. Accordingly, the greater regenerative performance of the assembly implant cannot be attributed specifically to β‐TCP spatial arrangement. Future studies should systematically vary β‐TCP distribution while maintaining comparable material content, porosity, geometry, and mechanical characteristics, preferably in larger‐animal models that provide greater spatial design freedom. Similarly, because a composition‐ and release‐matched β‐TCP‐coated porous Ti control was not included, the present study does not establish that modular integration is intrinsically superior to appropriately engineered surface bioactivation.

Finally, all implant configurations were evaluated within a plate‐stabilized defect model, and plate–implant load sharing was not directly quantified. The current mechanical results therefore demonstrate comparative structural behavior under the prescribed experimental and computational conditions rather than independent physiological load‐bearing capacity. Future studies incorporating fixation‐inclusive biomechanical analyses, degradation‐dependent mechanical testing, and clinically relevant loading conditions will be required to further define the mechanical contribution of the modular implant.

In summary, these limitations affect the quantitative generalization and mechanistic attribution of the present findings, but do not alter the central proof‐of‐concept: experimentally characterized spatial biological responses can be translated into a model‐informed strategy for organizing structural and bioactive modules within an implant. This framework shifts bioactive implant design from consideration of material composition alone toward the spatial integration of biological function within the regenerative space.

## 3 Conclusion

This study presents a bioadaptive modular implant strategy that integrates structural reinforcement with spatially localized bioactive regulation for large segmental bone repair. Using a simplified ECM‐inspired GelMA model, we identified distance‐dependent β‐TCP‐associated calcium distributions together with corresponding position‐dependent hBMSCs proliferation and osteogenic responses. The proximal region exhibiting the strongest and most consistent biological responses was subsequently used as a model‐derived spatial reference for organizing β‐TCP‐containing modules within a porous Ti alloy architecture.

In a plate‐stabilized rabbit femoral segmental defect model, the Ti alloy/β‐TCP assembly implant exhibited greater bone formation and bone ingrowth than the corresponding controls at the investigated time points. Newly formed bone and osteogenic activity were preferentially associated with β‐TCP‐containing regions and extended into adjacent porous Ti structures. These outcomes likely reflect the coupled contributions of structural reinforcement, β‐TCP‐mediated osteoconduction, and localized biochemical regulation. Collectively, the present study establishes a proof‐of‐concept for a model‐informed implant design strategy in which experimentally characterized spatial biological responses are translated into the spatial organization of structural and bioactive modules, providing a framework for the spatially informed design of regenerative implants.

## 4 Materials and Methods

### 4.1 Study Design

A 5% (w/v) GelMA hydrogel was used as a simplified ECM‐inspired, diffusion‐confined three‐dimensional model to investigate the spatial distribution of β‐TCP‐associated calcium species [51, 52, 53, 54, 55, 56, 57, 58, 59] and corresponding position‐dependent hBMSCs responses. The model was designed to suppress bulk convection and facilitate spatial analysis rather than to quantitatively reproduce native bone extracellular matrix. Based on the spatial responses identified in this model, a porous Ti alloy/β‐TCP modular implant was designed, with porous Ti alloy providing structural reinforcement and β‐TCP serving as a localized osteoconductive and bioactive component. Implant performance was subsequently evaluated in a rabbit femoral segmental defect model (Figure 1).

### 4.2 Preparation and Characterization of GelMA Hydrogel

GelMA with a degree of methacrylation of 60% was dissolved in phosphate‐buffered saline (PBS) containing lithium phenyl‐2,4,6‐trimethylbenzoylphosphinate (LAP) photoinitiator to prepare a 5% (w/v) solution. This formulation provided sufficient structural integrity while permitting cell encapsulation and diffusion‐mediated solute transport [60, 61, 62] and was not intended to represent an optimized or quantitatively bone‐equivalent matrix. Rheological measurements were performed using a rheometer (MCR 702e, Anton Paar, Germany) at 37.0 ± 0.1°C over shear rates of 10^−^^3^‐10^3^ s^−^^1^, and the viscosity, shear modulus, and mechanical properties were characterized.

### 4.3 Spatial Calcium‐Associated Distribution and Total Calcium Measurement

A three‐dimensional GelMA‐based diffusion‐confined model was established to characterize the spatial distribution of β‐TCP‐associated calcium. Briefly, 10 mL of GelMA hydrogel was placed in a 35‐mm culture dish, and a 3D‐printed porous β‐TCP sample was centrally embedded and photocrosslinked under 405‐nm light (5 W) for 10 s (Figure 2c). After the addition of 10 mL physiological saline, samples were incubated at 37°C for 3, 5, and 7 days. Alizarin Red S staining (ARS; pH 4.2, Beyotime, China) was used to qualitatively visualize the spatial distribution of calcium‐associated material within the GelMA matrix. For quantitative analysis, samples were collected from predefined regions at different distances from the β‐TCP source, and total elemental calcium was measured by inductively coupled plasma optical emission spectrometry (ICP‐OES; ICAP 7000 Series, Thermo Fisher Scientific, USA). Thus, ARS staining was used for qualitative spatial visualization, whereas ICP‐OES was used to quantify total calcium within the predefined regions. The measurements were performed under controlled saline‐based conditions and were used to characterize spatial calcium‐associated transport rather than local free Ca^2^^+^ activity.

### 4.4 FEA of Calcium Transport

Calcium transport within the GelMA matrix was simulated using COMSOL Multiphysics 5.6. A tetrahedral mesh was generated, and mesh independence was verified. The simulation duration was 7 days. Parameters included a calcium concentration of 2.56 mol/m^3^, temperature of 310.15 K, diffusion coefficient of 2.5 × 10^−^^10^ m^2^/s [63], and calcium flux of 2.1 × 10^−^^7^ mol/m^2^s, calculated from cumulative ICP‐OES release and exposed β‐TCP surface area. The β‐TCP/GelMA interface was defined as a constant‐flux boundary and the external boundaries as no‐flux boundaries. Calcium transport was described by:

$$\;\frac{\partial C}{\partial t}=\;D\nabla^{2}C$$

The simulated spatial profile was compared qualitatively with the experimentally measured calcium distribution.

### 4.5 Cell Culture and Seeding Within the Three‐Dimensional GelMA System

hBMSCs (Cyagen, USA; passage < 5) were used for all cell experiments. Porous materials were sterilized by autoclaving followed by ultraviolet irradiation for 30 min. Unless otherwise specified, hBMSCs were resuspended in sterile GelMA solution at 37°C and encapsulated at a density of 5 × 10^6^ cells per well, followed by photocrosslinking under 405‐nm light for 10 s. After gelation, the constructs were gently washed with PBS and cultured in complete medium, which was replaced every 48 h. Assay‐specific seeding densities are described in the corresponding experimental sections below.

### 4.6 Evaluation of Position‐Dependent Proliferation of hBMSCs

hBMSCs were seeded at a density of 1 × 10^6^ cells per well, and cell proliferation and viability in regions 1–3 (Figure 2d) were evaluated at days 3 and 7 using CCK‐8 assays (*n* = 6) and Calcein‐AM/PI live/dead staining (*n* = 6). For CCK‐8 analysis, 300 µL of 10% CCK‐8 working solution (Dojindo, Japan) was added to each sample and incubated at 37°C for 2 h. Subsequently, 100 µL of the reaction solution was transferred to a 96‐well plate, and absorbance was measured at 450 nm. For live/dead staining, samples were incubated with 1 mL of Calcein‐AM/PI staining solution (Beyotime, China) at 37°C in the dark for 30 min, washed with PBS, and imaged using an inverted fluorescence microscope (Axio Observer 7, Zeiss, Germany).

### 4.7 Evaluation of Position‐Dependent Adhesion of hBMSCs

Cell morphology and adhesion in different spatial regions were evaluated by F‐actin/DAPI staining after 3 and 7 days of culture (*n* = 6). Samples were fixed with 4% paraformaldehyde for 20 min, permeabilized with 0.1% Triton X‐100, and incubated with Actin‐Tracker working solution (Beyotime, China) for 60 min at room temperature in the dark. Nuclei were subsequently stained with DAPI for 5 min, and fluorescence images were acquired using an inverted fluorescence microscope.

### 4.8 Analysis of Position‐Dependent Osteogenic Differentiation

The expression of osteogenic genes (Runx2, ALP, OPN, and OCN) and Wnt/β‐catenin‐related genes (β‐catenin, GSK‐3β, Wnt1, Wnt3a, LEF, and TCF) was analyzed by quantitative reverse transcription polymerase chain reaction (qRT‐PCR, QuantStudio 6, Thermo Fisher Scientific, USA) after 7 and 14 days of culture (*n* = 6). Equivalent volumes of GelMA samples were lysed with 500 µL GelMA lysis buffer at 4°C for 30 min and centrifuged at 12 000 rpm for 5 min to collect cells. Total RNA was extracted and reverse‐transcribed for qRT‐PCR analysis. Gene expression was normalized to glyceraldehyde‐3‐phosphate dehydrogenase (GAPDH) and calculated using the 2^−ΔΔCt^ method.

### 4.9 β‐Catenin Immunofluorescence Staining in Different Spatial Regions

β‐catenin immunofluorescence staining was performed to evaluate position‐dependent differences in Wnt/β‐catenin‐related signaling (*n* = 6). GelMA samples were fixed with 4% paraformaldehyde for 20 min, permeabilized with 0.1% Triton X‐100 for 10 min, and blocked with 5% bovine serum albumin (BSA, Sigma) for 60 min. Samples were incubated with a primary antibody against β‐catenin (Beyotime, China) overnight at 4°C, followed by a fluorophore‐conjugated secondary antibody for 2 h at room temperature in the dark. Fluorescence images were acquired using an inverted fluorescence microscope, and relative fluorescence intensity was quantified using ImageJ.

### 4.10 Gene Expression Profiling

To investigate global transcriptional changes associated with the position‐dependent β‐TCP‐associated microenvironment, gene expression profiling was performed using the Agilent microarray platform. hBMSCs were seeded into the three‐dimensional GelMA culture system at a density of 1.5 × 10^5^ cells per well. After 7 days of culture, cells were collected and lysed using Trizol reagent (Invitrogen, USA). For each experimental group, cell lysates from eight independent wells were pooled into cryogenic screw‐cap tubes (−192°C compatible) to ensure sufficient RNA yield, with the total number of collected cells exceeding 1 × 10^6^ cells per sample. Total RNA was extracted according to the manufacturer's instructions. RNA quality was assessed before hybridization and subsequent mRNA expression profiling using the Agilent microarray platform. Differentially expressed genes were identified and analyzed according to the manufacturer's standard protocol. Bioinformatics analysis was subsequently performed to identify biological pathways associated with the position‐dependent cellular responses within the β‐TCP‐associated microenvironment, with particular attention paid to Wnt/β‐catenin‐related signaling pathways involved in osteogenic differentiation.

### 4.11 Wnt/β‐Catenin Signaling Inhibition and Western Blot Analysis

To examine the involvement of Wnt/β‐catenin signaling in the position‐dependent osteogenic response, hBMSCs were treated with 2 µM XAV939 (Selleck, USA) for 24 h and subsequently cultured in fresh XAV939‐containing medium for 7 days. For Western blot analysis, GelMA samples were washed three times with PBS (3 min each). Equivalent volumes of hydrogel samples were collected and transferred into centrifuge tubes. Cell lysis buffer was added, and samples were incubated at room temperature for 30 min. The lysates were centrifuged at 12 000 rpm for 10 min, and the collected supernatants were subjected to protein denaturation at 100°C for 5 min. Equal amounts of protein samples (20 µL) were loaded onto sodium dodecyl sulfate–polyacrylamide gel electrophoresis (SDS‐PAGE gels) and electrophoresed at 130 V for 70 min. Proteins were subsequently transferred onto methanol‐activated polyvinylidene fluoride (PVDF) membranes at 110 V for 80 min. The membranes were blocked with 1% BSA for 60 min at room temperature and incubated overnight at 4°C with primary antibodies. After washing three times with phosphate‐buffered saline containing Tween 20 (PBST), membranes were incubated with horseradish peroxidase (HRP)‐conjugated secondary antibodies for 40 min at room temperature. Protein signals were visualized using enhanced chemiluminescence (ECL), and band intensities were quantified using ImageJ software.

### 4.12 Design of the Assembly Implant

Rabbit femoral geometry was reconstructed from micro‐CT data to design anatomically matched implants. According to ASTM F2721‐09 and YY/T 1679–2021, a mid‐diaphyseal segmental defect with a length of 1.61 times the maximum femoral cross‐sectional diameter was established (Figure S15a–d). The mid‐diaphysis was selected because of its predominantly cortical and mechanically demanding environment, limited cancellous bone contribution, and suitability for standardized osteotomy, fixation, and spatial bone‐ingrowth analysis. Three implant configurations were designed: porous Ti alloy, porous Ti alloy/β‐TCP modular assembly, and porous β‐TCP implants. The porous Ti alloy module consisted of a bowstring‐arch lattice [64] with 75% porosity, a 1‐mm unit size, an average pore diameter of approximately 600 µm, and a compressive strength of 260 MPa. The β‐TCP module had 65% porosity, a 1.4‐mm unit size, and an average pore diameter of approximately 550 µm. Because the strongest cellular responses in the GelMA model were observed within region 1 (1–4 mm from the β‐TCP source), approximately 4 mm was used as a model‐derived spatial design reference, rather than a biological threshold or universal design criterion. Accordingly, adjacent porous Ti regions were maintained within approximately 4 mm of the β‐TCP modules. The modular components were stabilized using a Ti alloy connector with four 0.8‐mm embedded clasps (Figure 4a and Figure S5). A customized 40‐mm‐long, 0.6‐mm‐thick fixation plate containing six 2.5‐mm screw holes was used to maintain anatomical alignment and defect stability.

### 4.13 Fabrication and Post‐Processing of Implants

Ti‐6Al‐4 V components were fabricated by selective laser melting (SLM) under argon using a laser power of 160 W, scanning speed of 1000 mm/s, spot diameter of 0.06 mm, and layer thickness of 30 µm. Printed components were heat‐treated at 800°C for 120 min and sandblasted at 0.4 MPa for < 10 s to remove residual surface particles (Figure S16a,b). Surface morphology, roughness, extract cytotoxicity, compressive properties, hardness, and wear resistance were subsequently evaluated (Figure S17). β‐TCP components were fabricated by digital light processing (DLP) using a 50 vol% slurry, 50‐µm printing layers, and 90 W UV irradiation. The initial and subsequent layer exposure times were 74 and 7 s, respectively. Printed components were heated to 500°C for 5 h for binder removal and subsequently sintered at 1050°C for 2 h (Figure S16c). The process followed YY/T 0683–2008, YY/T 1558.3‐2017, and ISO 13175‐3:2012.

### 4.14 FEA of Implant Mechanical Behavior

FEA was performed using ANSYS Workbench 17.0. The model included the femur, fixation plate, screws, Ti connector, porous Ti alloy, and β‐TCP modules, which were assumed to be isotropic, homogeneous, and linearly elastic. The elastic modulus was set to 3, 5, and 8 GPa, respectively for cortical bone, porous Ti alloy, and porous β‐TCP, with Poisson's ratios of 0.30, 0.36, and 0.32, respectively. The experimentally determined compressive strengths of porous Ti alloy and β‐TCP were 260 and 80 MPa, respectively [64]. A compressive stress of 230 MPa was applied. Approximately five million tetrahedral elements were generated with local mesh refinement at implant interfaces, and identical contour scales were used for comparison among implant configurations.

### 4.15 Compressive Fatigue Performance Testing of Implants

Compressive fatigue testing was performed using an Instron E3000 system (*n* = 6). Polyoxymethylene (POM) resin was used solely as a standardized support fixture to provide consistent boundary conditions for comparative testing and was not intended to reproduce the mechanical behavior of cortical bone (Figure S18). Pristine, non‐degraded implants were cyclically loaded between 100 and 1000 N at R = 0.1 and 10 Hz until structural failure, deformation, loosening, or 10^6^ cycles. The results therefore represent the initial comparative fatigue resistance of the implant configurations rather than long‐term fatigue behavior after degradation.

### 4.16 Design of Personalized Surgical Guides

Personalized positioning and osteotomy guides were designed from reconstructed femoral anatomy to facilitate reproducible defect localization, osteotomy, implant placement, and anatomical alignment (Figure S7). The positioning guide contained six screw channels and surface grooves for stable fixation, whereas the osteotomy guide incorporated femur‐matching grooves, Kirschner‐wire channels, and a locally reinforced cutting surface to define reproducible osteotomy planes.

### 4.17 Animal Allocation, Blinding, Attrition, and Humane Endpoints

No formal prospective power calculation was performed because this was an exploratory in vivo study. Three implant configurations were evaluated at 4, 8, and 12 weeks, resulting in nine experimental groups. Five evaluable rabbits were predefined for each group (*n* = 5), corresponding to 45 planned experimental animals. In addition, one reserve rabbit was assigned to each group, resulting in nine reserve animals and a total of 54 rabbits within the experimental allocation framework. The 45 planned experimental rabbits initially underwent the corresponding surgical procedures. During the study, five animals died because of severe infection (*n* = 2), femoral fracture (*n* = 2), or severe diarrhea (*n* = 1). Five of the nine reserve rabbits were subsequently used as replacements in the corresponding groups and underwent the same experimental procedures. The remaining four reserve rabbits were not subjected to surgery and were not included in the analysis. Thus, a total of 50 rabbits underwent experimental procedures, and 45 animals reached their scheduled endpoints and were included in the final analyses, maintaining *n* = 5 evaluable animals per group. Animals were screened, identified, and allocated to the predefined groups by animal‐facility personnel independently of the operating surgeon, and surgeries were conducted using a prespecified rotating sequence among implant groups. Surgeon blinding was not feasible because the implant configurations were macroscopically distinguishable; however, Micro‐CT, histological, and quantitative analyses were performed by investigators blinded to group allocation. Animals were monitored daily, and predefined humane endpoints included > 20% body‐weight loss, inability to eat or drink, severe respiratory distress, paralysis, or persistent uncontrolled pain or distress.

### 4.18 Surgical Procedure and Postoperative Care

All animal experiments were conducted in accordance with relevant regulations, institutional guidelines, and the approved animal experimental protocol. The experimental procedures were reviewed and approved by the Animal Care and Experiment Committee of Guangzhou Huateng Biomedical Co., Ltd. (Approval No. C202304‐5). The study was additionally filed with South China University of Technology and conducted under its institutional oversight. Before surgery, general anesthesia was induced by intravenous administration of 5% pentobarbital sodium at 0.4 mL/kg (20 mg/kg) through the marginal ear vein. After an adequate depth of anesthesia was confirmed, the surgical area was shaved and disinfected. A longitudinal incision was made along the intermuscular plane between the rectus femoris and vastus lateralis to minimize muscle disruption and avoid the major femoral vessels and nerves. The femur was exposed using retractors and curved forceps. The customized positioning guide was placed on the femur to define the predefined defect location, and Kirschner wires were inserted through the guiding channels to identify the proximal and distal osteotomy positions. After removal of the positioning guide, the osteotomy guide was sequentially placed at the proximal and distal positions, and osteotomies were performed using an oscillating saw to create the segmental femoral defect. The customized implant was subsequently inserted into the defect, and the fixation plate was secured using locking screws. The K‐wire holes were enlarged to 1.5 mm where required for final screw fixation. After fixation, the muscle and skin layers were sutured sequentially. The surgical site was disinfected with povidone‐iodine, covered with sterile gauze, and protected using a polymer bandage to minimize postoperative wound disturbance (Figure S6). Postoperative analgesia consisted of 2% lidocaine hydrochloride (20 mg/kg, intramuscularly, once daily for 5 days), and sodium penicillin (400 000 IU, intramuscular, once daily for 5 days) was administered prophylactically.

### 4.19 Sample Harvest and Micro‐CT Analysis

Animals were euthanized at 4, 8, or 12 weeks according to the approved protocol. Femora harvested at 4 and 12 weeks were scanned using a Quantum GX2 system at 90 kV, 88 µA, 110 mGy, 1.0‐mm Cu filtration, 144‐µm voxel size, and a scanning time of 4 min. Quantitative analysis was performed within a cylindrical region of interest (ROI) spanning the original defect and implant‐associated porous region between the proximal and distal osteotomy planes. No dedicated metal‐artifact‐reduction algorithm was applied. Reconstructed datasets were processed using Mimics 21.0 with fixed attenuation ranges of 3100–4200 HU for Ti alloy, 1500–1800 HU for residual β‐TCP, and 225–1304 HU for mineralized bone. The β‐TCP mask was further corrected according to its predefined scaffold geometry and spatial location, after which Ti and β‐TCP masks were excluded before bone‐volume quantification. Identical ROI definitions, thresholds, and segmentation criteria were applied to all specimens. BV/TV was calculated from the segmented mineralized‐bone mask. Because partial‐volume effects and attenuation overlap between bone and residual β‐TCP could not be completely eliminated, particularly at bone–ceramic interfaces, BV/TV was interpreted as an estimate of mineralized bone volume and evaluated together with undecalcified histology. Representative reconstructed and segmented slices are shown in Figure S19. A formal intra‐ or inter‐observer reliability analysis was not prospectively performed; therefore, fixed thresholds and standardized geometry‐constrained segmentation criteria were applied consistently to minimize subjective adjustment.

### 4.20 Histological and Immunohistochemical Analysis

Femoral specimens harvested at 4 and 12 weeks were used for undecalcified histological analysis. The specimens were fixed in 4% paraformaldehyde, dehydrated, and embedded in methyl methacrylate without decalcification. Longitudinal sections parallel to the femoral axis and fixation plate were prepared and polished to approximately 5 µm (Figure S20). Methylene blue/basic fuchsin (MB/BF) and Masson's trichrome staining were used to evaluate mineralized bone formation and matrix deposition, respectively. For MB/BF staining, sections were hydrated and stained with 0.02% methylene blue solution for 3 min at room temperature. After differentiation with hydrochloric acid, ethanol, and sodium hydroxide solutions, sections were stained with 0.1% basic fuchsin solution for 1 min, dehydrated, mounted, and imaged. For Masson's trichrome staining, hydrated sections were stained with hematoxylin for 5 min, differentiated, and rinsed with water. Subsequently, sections were stained with Ponceau and toluidine blue solutions for 6 min each, followed by differentiation using glacial acetic acid and phosphotungstic acid solutions. After dehydration, sections were mounted and imaged. For immunohistochemical analysis, femoral specimens harvested at 8 weeks were used to evaluate ALP and β‐catenin expression. Sections were subjected to antigen retrieval and incubated with primary antibodies against ALP and β‐catenin, followed by secondary antibodies and 3,3′‐diaminobenzidine (DAB) visualization. Histological and immunohistochemical analyses were independently evaluated by two investigators blinded to group allocation.

### 4.21 Quantitative Histological Analysis of Bone‐Ingrowth Depth

Longitudinal undecalcified sections were used for quantitative spatial analysis. Overall bone‐ingrowth depth was defined as the maximum longitudinal penetration of newly formed bone from the host bone–implant interface into the porous implant. For Ti alloy/β‐TCP implants, the β‐TCP/Ti boundary was additionally used as a spatial reference, and the extension of newly formed bone from β‐TCP‐containing regions into adjacent porous Ti was measured longitudinally. At least three sections per specimen were analyzed and averaged to generate one specimen‐level value for statistical analysis.

### 4.22 Statistical Analysis

Statistical analyses were performed using GraphPad Prism 9.5. Comparisons among more than two groups at each predefined time point were performed independently using one‐way ANOVA followed by Tukey's multiple‐comparison test. Comparisons between two independent groups were performed using an unpaired two‐tailed Student's *t*‐test. No inferential comparisons were performed across different time points unless otherwise specified. The exact biological replicate number (*n*) is provided in each figure legend. Data are presented as mean ± SD. Statistical significance was defined as *p* < 0.05, with ^*^ *p* < 0.05, ^**^ *p* < 0.01, and ^***^ *p* < 0.001.

## Author Contributions

**Heng Zhang**: conceptualization, methodology, software, data curation, investigation, Writing – review and editing, Writing – original draft, validation, formal analysis. **Jingjing Diao**: methodology, supervision, investigation, data curation, formal analysis. **Junhua Ke**: conceptualization, software. **Naru Zhao**: conceptualization, supervision, funding acquisition. **Yudi Kuang**: funding acquisition, writing – review and editing, validation, conceptualization, formal analysis. **Yingjun Wang**: funding acquisition, supervision, conceptualization.

## Conflicts of Interest

The authors declare no conflicts of interest.

## Supporting information




**Supporting File**: advs78161‐sup‐0001‐SuppMat.docx.

## Data Availability

The data that support the findings of this study are available from the corresponding author upon reasonable request.
