# Supplementary material for: Engineering Spatially Localized Bioactive Microenvironments in Assembly Implants for Spatially Extended Bone Ingrowth
Source: Adv Sci (Weinh). 2026 Oct 4:e78161. Online ahead of print. doi: 10.1002/advs.78161 (PMC13634580; doi:10.1002/advs.78161)
Supplement: Supplementary file 1 [file ADVS-9999-e78161-s001.docx]

**Supporting Information**

**Engineering Spatially Localized Bioactive Microenvironments in Assembly Implants for Spatially Extended Bone Ingrowth**

Heng Zhang, Jingjing Diao, Junhua Ke, Naru Zhao*, Yudi Kuang*, and Yingjun Wang*

H. Zhang, J. Ke, N. Zhao, Y. Wang

School of Materials Science and Engineering, South China University of Technology, Guangzhou, 510641, PR China

E-mail: mshengzhang@163.com

H. Zhang, J. Ke, N. Zhao, Y. Wang

National Engineering Research Center for Tissue Restoration and Reconstruction, South China University of Technology, Guangzhou, 510006, PR China

J. Diao

Medical Devices Research & Testing Center of SCUT, Guangzhou, 510006, PR China

Y. Kuang, Y. Wang

Guangdong Institute of Advanced Biomaterials and Medical Devices, Guangzhou, 510535, PR China

Y. Wang

School of Biomedical Science and Engineering, South China University of Technology, Guangzhou International Campus, Guangzhou, 511442, PR China

**Figure S1.** Mechanical characterization of GelMA hydrogel. a. Viscosity-shear rate curve. b. Tensile stress-strain curve. c. Shear modulus-shear rate curve.

**Figure S2.** Cell proliferation and spreading statistics: a. Cell number and fluorescent area. b. Cell fluorescent staining spreading area. Statistical comparisons were performed independently at each time point using one-way ANOVA followed by Tukey’s multiple-comparison test. Data are presented as mean ± SD, n = 6.

**Figure S3**. a. Expression of Wnt/β-catenin pathway target genes (LEF and TCF) at different predefined spatial regions. b. Quantitative histograms of β-catenin-DAPI immunofluorescence staining fluorescent area. Statistical comparisons were performed independently at each time point using one-way ANOVA followed by Tukey’s multiple-comparison test. Data are presented as mean ± SD, n = 6. **p* < 0.05, ***p* < 0.01, and ****p* < 0.001.

**Figure S4.** Normalized spatial biological responses used to derive the model-informed design reference. Region 1 (1–4 mm from the β-TCP source) exhibited the strongest and most consistent biological responses among the predefined sampling regions. The approximately 4-mm value corresponds to the outer boundary of region 1 and is used as an empirical design-guiding range rather than a formally determined biological threshold.

**Figure S5.** a. Schematic of 3D-printed assembly implants. b. Schematic of the assembly for 3D-printed implants. c. Views of the 3D-printed assembly implants.

**Figure S6.** Surgical workflow for the rabbit large segmental femoral defect model.

**Figure S7.** Design diagrams of the surgical positioning guide, osteotomy guide, and formulation of the precise surgical plan.

**Figure S8.** X-ray images immediately after surgery and at 12 weeks.

**Figure S9.** Gross appearance of retrieved specimens at 4 weeks and 12 weeks post-implantation.

**Figure S10.** Quantitative histomorphometric analysis of newly formed bone based on MB/BF and Masson staining. Statistical comparisons were performed independently at each time point using one-way ANOVA followed by Tukey’s multiple-comparison test. Data are presented as mean ± SD, n = 5.

**Figure S11.** MB-BF staining at 4 and 12 weeks post-implantation, showing more details corresponding to **Figure 5c**.

**Figure S12.** Masson staining at 4 and 12 weeks post-implantation, showing more details corresponding to **Figure 5d**.

**Figure S13.** Spatial analysis of bone extension from β-TCP-containing regions into adjacent porous Ti regions. a-b. Schematic illustrations of the Ti alloy/β-TCP assembly configurations. c-d. Representative MB-BF-stained longitudinal sections at 12 weeks, showing newly formed bone extending from β-TCP-containing regions into adjacent porous Ti regions. e. Quantification of bone-extension distance in the Ti alloy (70%)/β-TCP (30%) configuration (n = 5). The β-TCP/Ti interface was defined as 0 mm, and the distance to the furthest continuous newly formed bone within the adjacent porous Ti region was measured longitudinally. Isolated bone islands were excluded. Three longitudinal sections were analyzed per rabbit, and their mean was used as the specimen-level value. Quantitative depth analysis was not performed for the Ti alloy (30%)/β-TCP (70%) configuration because the adjacent porous Ti regions were only approximately 2–3 mm in length, which geometrically constrained the measurable extension distance.

**Figure S14.** Exploratory histological comparison of an alternative 50/50 Ti alloy/β-TCP assembly configuration at 4 and 12 weeks. In this configuration, porous Ti alloy was positioned peripherally and β-TCP centrally. Representative MB/BF- and Masson-stained longitudinal sections show that newly formed bone was associated with β-TCP-containing regions and extended toward adjacent porous Ti structures. This configuration was exploratory and was not prospectively matched to the other configurations for composition, geometry, interface area, or mechanical properties.

**Figure S15.** Schematic illustration of the large segmental bone defect model and implant designs. a. Large segmental femoral defect model. b–d. Individually designed 3D-printed porous β-TCP implant, Ti alloy/β-TCP assembly implant, and porous Ti alloy implant for the corresponding defect site, respectively.

**Figure S16.** Fabrication and post-processing images of implants. a. As-printed Ti alloy module morphology and its microstructure. b. Sand-blasted Ti alloy module morphology and its microstructure. c. 3D printed porous β-TCP module and its microstructure.

**Figure S17.** Effects of sandblasting on the surface characteristics, cytocompatibility, scratch response, wear behavior, and compressive properties of porous Ti alloy. a. Representative surface-roughness profiles before and after sandblasting. b. Cell viability of samples with and without sandblasting at different culture times. c. Nanoscratch load–depth response and representative indentation morphology. d. Scratch-force/area profiles and representative scratch tracks. e. Compressive stress–strain curves of samples with and without sandblasting. Data are presented as mean ± SD, n = 6 where applicable.

**Figure S18.** Compression-fatigue testing setup and comparative fatigue performance of the implant configurations under standardized in vitro loading conditions. POM was used as a standardized support fixture, and pristine, non-degraded implants were tested (n = 6 per configuration).

**Figure S19.** Representative Micro-CT reconstruction and segmentation workflow used for quantitative analysis. Reconstructed slices and corresponding segmented images are shown for porous Ti alloy, Ti alloy/β-TCP assembly, and porous β-TCP specimens. Ti alloy and residual β-TCP were identified using predefined attenuation ranges and geometry-constrained segmentation, and the corresponding masks were excluded before quantification of mineralized bone within the predefined ROI. The same thresholds and segmentation criteria were applied to all specimens.

**Figure S20.** Schematic of sectioning positions.
